# The Interplay Between Household Risk Perception of Parasitic Infections and Water, Sanitation, and Hygiene (WASH) Practices: Evidence From an Urban Poor Community in the Philippines

**DOI:** 10.7759/cureus.57532

**Published:** 2024-04-03

**Authors:** Ryan V Labana, Ma. Cate Nicole M Borda, Ryan Toribio A Campo, Maria Antonia V Ocampo

**Affiliations:** 1 Center for Integrated Community Science Research, Research Institute for Science and Technology, Polytechnic University of the Philippines, Manila, PHL; 2 Department of Biology, College of Science, Polytechnic University of the Philippines, Manila, PHL

**Keywords:** soil-transmitted helminths, food and waterborne diseases, baseco compound, community health research, health risk behavior, parasitic disease, urban health determinants, neglected (re)emergent tropical disease

## Abstract

Background: Parasitic diseases pose challenges in impoverished urban settlements with limited access to clean water, proper hygiene, and sanitation (WASH). This study assesses WASH practices and risk perceptions of parasitic infections among households in the Bataan Shipyard and Engineering Corporation (BASECO) Compound in Manila, an urban poor community in the Philippines.

Methods: A cross-sectional study design was employed to collect data through a self-administered questionnaire. Descriptive statistical analysis was performed to assess the sociodemographic profile, household WASH practices, and respondents’ risk perception of parasitic infections. Linear regression analysis was utilized to examine the relationship between these variables.

Results:A survey was conducted with 363 households, of which 237 (65.3%) used distilled and purified water from the water refilling stations in the community for drinking. Meanwhile, 120 households (33.10%) consumed tap water. Boiling water was a commonly used method (n=146; 56.60%) for treating drinking water. Most households had flush toilets with septic tanks (n=244; 67.20%), water sources for handwashing (n=307; 84.57%) and soap for handwashing (n=356; 98.10%). On average, they washed their hands 6-10 times daily (n=159; 43.80%). Most households were aware that drinking untreated water (n=318; 87.6%), improper food washing (n=309; 85.1%), using contaminated water sources (n=301; 82.9%), and consuming raw or undercooked meat (n=298; 82.1%) could lead to parasitic infections. 316 respondents (87.1%) identified diarrhea as the most common symptom of parasitic infection. Relationships were found between access to drinking water and the number of household members (*B*=0.191; p-value=0.001), personal hygiene and the respondents' knowledge of parasitic infections (*B*=0.112; p-value=0.047), and the overall WASH score with household income (*B*=0.105; p-value=0.045).

Conclusions: The WASH conditions in BASECO, Manila need improvement. Factors associated with their WASH practices include risk perception of parasitic diseases, socioeconomic disparity, and household overcrowding. These factors play a crucial role in identifying areas for improvement and promoting health policies for urban poor communities in the Philippines.

## Introduction

Parasitic diseases are often transmitted in impoverished rural regions and indigenous communities with limited access to healthcare services and poor water, sanitation, and hygiene (WASH) practices [1,2]. However, the incidence of parasitic diseases also increases in urban areas due to urbanization and globalization. These factors result in changing interactions between humans, animals, and the environment, quickly transmitting parasites [3]. Some of these parasites come from rural communities and are brought to urban settings through human migration. Moreover, rapid urban expansion jeopardizes access to sufficient water supplies, housing, and infrastructure, leading to a decline in proper WASH practices. As a result, public health experts consider urban areas potential hotspots for transmitting parasitic diseases due to their diverse and densely populated nature [4].

An estimated 4.2 billion people lived in urban areas in 2018, accounting for 55% of the global population. The overcrowding of people in urban areas and the increasing number of impoverished urban settlements worsen the delivery of WASH services among low-income families. Consequently, many vulnerable individuals reside in urban poor communities [5]. Poor WASH practices can lead to the contraction of diseases, including parasitic infections, causing food and waterborne illnesses (FWBDs). These illnesses remain a significant public health concern in many countries [6]. FWBDs account for 80% of all illnesses in developing nations, straining healthcare systems and hindering socioeconomic development [7]. They are closely linked to exposure to, and consumption of water contaminated with pathogens [8]. FWBDs include historically significant diseases such as cholera, diarrhea, and typhoid fever, all of which have been significant causes of death [9].

Soil-transmitted helminthiasis is another common problem associated with poor WASH practices. The most prevalent soil-transmitted helminth is *Ascaris lumbricoides*, which infects approximately one billion people. Meanwhile, *Trichuris trichiura* and hookworms (*Necator americanus* and *Ancylostoma duodenale*) infect 600 to 800 million people yearly [10]. The Philippines is an endemic area for these parasitic infections, posing a significant public health concern for the country [11]. Hence, it is crucial to provide communities with sufficient knowledge and access to WASH facilities as preventive measures [12].

This public health issue is also a concern for many health organizations. UNICEF's urban WASH program promotes equitable access to WASH for urban poor communities, focusing on the welfare of children and their families [5]. In Ghana, the WASH for Urban Poor (WASH-UP) program aims to improve water supply and basic sanitation for the people and strengthen local governance for WASH [13]. In the Philippines, the Philippine Red Cross implemented a program in a densely populated urban poor community in Tondo, Manila, focusing on improving access to safe water and basic sanitation [14]. Despite the availability of WASH programs as an intervention effort against parasitic and other related infections in urban poor communities, there remains a significant gap in understanding the determinants of WASH practices in urban poor communities.

This study aims to assess the WASH practices of households in an urban poor community in Manila, Philippines. It also seeks to understand the households' perceptions of the risks associated with parasitic infections. Information on WASH practices and health risk perceptions in urban settings is crucial for establishing a solid foundation, identifying areas for improvement, and promoting health policies for the well-being of urban poor communities in the Philippines.

## Materials and methods

Study design

This study used a cross-sectional design to collect data at a specific point in time. Its main goal was to assess the risk perceptions of an urban poor community regarding the transmission of parasitic diseases and to evaluate their WASH practices. We collected data through a self-administered questionnaire, with respondents providing answers based on their level of certainty.

Study area

The study was conducted in Barangay 649, Zone 68, also called the Bataan Shipyard and Engineering Corporation (BASECO) Compound, Manila, in the Philippines. BASECO Compound or BASECO is an urban slum adjacent to Manila Harbor, with the Pasig River on one side and a swamp on the other. It primarily consists of informal settlements, where residents need land titles and reside in makeshift buildings and homes with unorganized layouts. It has an official population of 59,847 people, as reported by the 2015 Census Population and Housing conducted by the Philippine Statistics Authority (PSA) [15]. With a population of around 60,000 residing in a 53-hectare area, the BASECO Compound is recognized as one of Manila's largest and most densely populated slums in the Philippines. This overcrowded community faces numerous challenges, including inadequate sanitation facilities and limited availability of safe and affordable drinking water [16].

Population, sample size, and sampling technique

**Figure 1:**
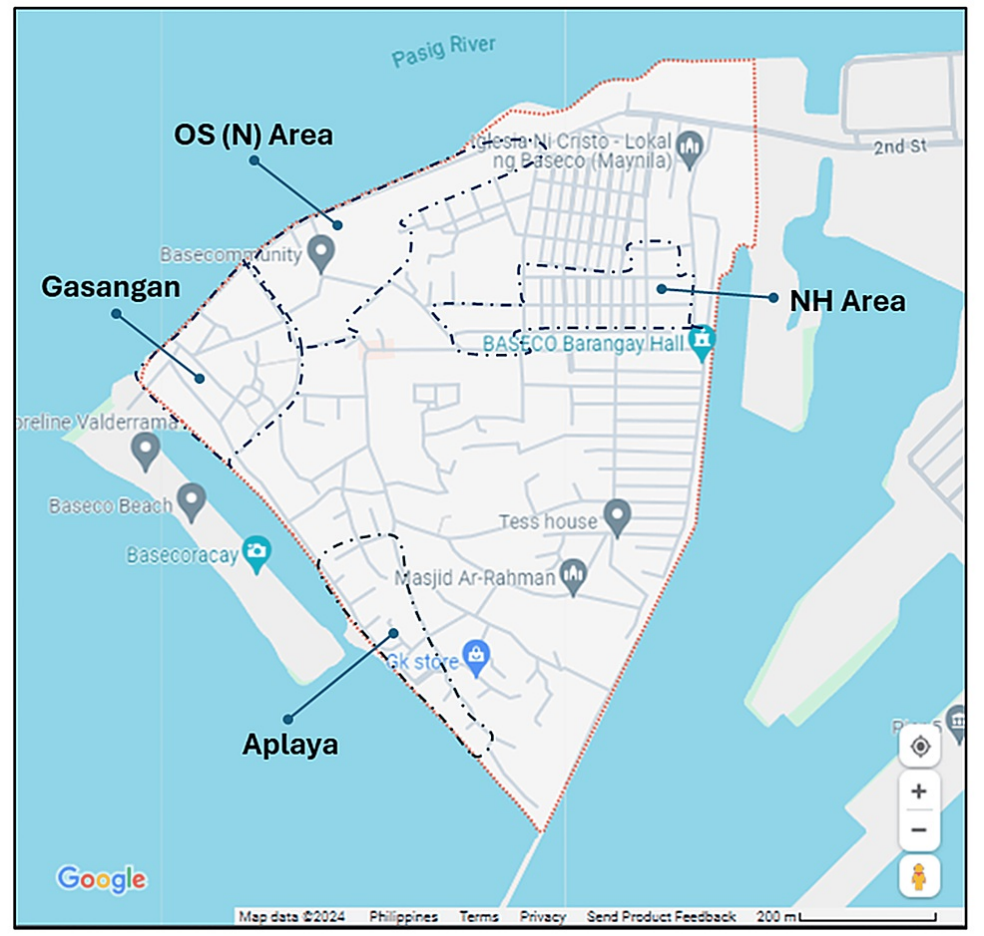
Google maps locations of sampling sites in BASECO, Manila, Philippines: NH Area, OS (N) Area, Gasangan, and Aplaya

According to the 2015 census, BASECO Compound has a population of 59,847 people living in 14,121 households. Each household consists of an average of four members. To determine the sample size, we referred to the urban morphology of BASECO Compound proposed by Cutini et al. [17], which divided BASECO into 14 strata. We calculated the difference in surface area among these strata using Google Earth Web. The percentage allocation for each stratum was determined by dividing its area by the total area of 14 strata. Based on the total number of households (n=14,121), we used proportional allocation to determine the number of households in each stratum. We purposively selected four strata for this study based on their accessibility and safety. Two out of the 14 strata in BASECO were excluded because they contained public buildings and infrastructure. In addition, certain strata could not be accessed by public transportation due to a narrow road system. Moreover, during our initial visits, we identified safety concerns in some strata. Out of the six strata that were accessible to the public via the primary road system and presented no safety issues, we selected only four to serve as the research site for this study. These four strata are the NH area with terraced units, OS (N) North area, Gasangan, and Aplaya, which contain a total of 3,663 households. Using Slovin's formula *n *= N / (1 + Ne^2^) [18], where the value of *N* is 3,663 and the margin of error (e) is 0.05, the sample size for the survey was determined to be 363. The sample size (n=363) was distributed among the four strata using proportional allocation based on their respective sizes. Within each stratum, we randomly selected households. The heads of households or individuals aged 18 years and above who resided in BASECO in the past three years represented the household in the survey. Figure 1 presents the map of BASECO with the sampling sites [19].

Survey instrument

The questionnaire began with an introduction that explained the study's objectives and provided completion instructions. The Informed Consent Form followed, which needed to be signed by the respondent to continue. We also collected sociodemographic data.

The questionnaire consisted of two main sections. The first section assessed the respondents' WASH practices using modified versions of the WHO/UNICEF JMP's Core Questions on WASH Surveys [20]. This section included questions about access to safe drinking water, household hygiene with a focus on toilet facilities, and personal hygiene with a focus on handwashing practices. The internal reliability of the WASH practices measures was best at Cronbach’s α 0.91. The questionnaire was subjected to content validation through the assessment of three experts. They evaluated the clarity (rated on a scale of 1-Not Clear to 4-Very Clear) and relevance (rated on a scale of 1-No Relevance to 4-Highly Relevant) of the questions while considering the underlying constructs and their dimensions. The average scale content validity index (S-CVI/Ave) was calculated as 1.

The second section of our survey followed the Standard Questionnaire on Risk Perception of an Infectious Disease Outbreak [21]. The Municipal Public Health Service Rotterdam-Rijnmond (GGD) and the National Institute for Public Health and the Environment (RIVM) in the Netherlands initially developed the questionnaire. In this section, we specifically targeted parasitic infections and asked participants about their knowledge, seriousness, and susceptibility perception. The internal reliability of the risk perception measures was better at Cronbach’s α 0.86, subjected to content validity (S-CVI/Ave = 1).

WASH indices

The WASH practices in BASECO, Manila were assessed using the indices developed by Jeyakumar et al. [22], with some modifications. These indices were based on the principal components of WASH programs in the community: drinking water availability, household hygiene, and personal hygiene [13,14].

The Drinking Water Index (DWI) was constructed based on four variables related to water accessibility, including the source of drinking water, drinking water treatment, sharing of water sources, and time to obtain water. The Household Hygiene Index (HHI) comprised three variables concerning household toilet facilities, including the type of toilet facility, sharing of toilet facility, and the location of the toilet. The Personal Hygiene Index (PHI) comprised three variables related to personal hygiene, including the availability of a handwashing station, the use of soap for handwashing, and the frequency of handwashing.

The WASH indices were measured based on the responses to the questionnaire. The WASH indicators standards [23] were used as a basis to determine whether the responses to the questionnaire implied a positive or negative observation of the WASH practices. A positive observation garnered a score of 1, while those that did not meet the standards garnered a score of 0. We calculated the indices by adding up the scores.

Ethical considerations

The study followed ethical principles aimed at promoting well-being and preventing harm. It adhered to the following principles: 1) obtaining informed consent from potential research participants; 2) ensuring no harm to the participants; 3) safeguarding their privacy, anonymity, and confidentiality; 4) refraining from employing misleading practices; and 5) allowing participants to withdraw from the study at any time. The research received ethics approval from the PUP Research Ethics Committee (UREC-2022-0162) on September 1, 2022, indicating compliance with the Philippine Health Research Ethics Board (PHREB) requirements.

Statistical treatment of data

The survey data were entered manually into Microsoft Excel 2021. Descriptive statistical analysis determined the respondents' sociodemographic profile, the WASH practices of households, and the participants' risk perception of parasitic infections. Linear regression analysis tested the relationships between sociodemographic characters, risk perception, and WASH practices. We performed the statistical analysis using SPSS (Version 25.0; IBM Corp., Armonk, NY).

## Results

Profile of the respondents

A total of 363 individuals participated in the study, representing the members of each household. Among the participants, the majority were female (n=258; 71.07%), and most fell into the 31-40 age group (n=102; 28.25%). Their highest level of education was typically high school (n=110; 30.30%). The households typically comprised 5-8 members (n=197; 54.27%). Most of the participants were unemployed at the time of the survey (n=109; 30.19%) and had a monthly income of PHP 10,000 or less (n=266; 73.68%). For a complete overview of the sociodemographic profiles of the respondents (Table 1).

**Table 1:** Sociodemographic profiles of the respondents

Sociodemographic Characteristics		f	%
Sex	Female	258	71.07
	Male	105	28.93
Age	18-20 y/o	25	6.93
	21-30 y/o	90	24.93
	31-40 y/o	102	28.25
	41-50 y/o	67	18.56
	51-60 y/o	55	15.24
	61-70 y/o	18	4.99
	70 and above	6	1.66
Educational Attainment	Elementary level	50	13.77
	Elementary graduate	31	8.54
	High School level	110	30.3
	High School graduate	77	21.21
	College level	40	11.02
	College Graduate	50	13.77
	Did not graduate from any level	5	1.38
Number of Household Members	1-4	114	31.4
	5-8	197	54.27
	9-12	42	11.57
	13-16	8	2.2
	17 and above	2	0.55
Occupation	Unemployed	109	30.19
	Housewife	93	25.76
	Vendor	37	10.25
	Driver	20	5.54
	Food Handler	13	3.6
	Others	91	25.07
Total Monthly Household Income	PHP 10, 000 and below	266	73.68
	PHP 10, 001 - 20, 000	70	19.39
	PHP 20, 001 - 30, 000	17	4.71
	PHP 30, 001 and above	2	0.55
	Did Not Disclose	8	2.22

WASH condition of households in BASECO, Manila

Table 2 presents the WASH conditions of households in BASECO, Manila. Regarding the source of drinking water, 237 households (65.3%) used distilled and purified water from the water refilling stations in the community for drinking. Meanwhile, 120 households (33.10%) consumed tap water. The safety of tap water, whether from surface or groundwater, depends on the integrity and management of the local water utility. Public health authorities recommend treating water from this source with a reliable water treatment [23]. Boiling water for treatment was the most common method used by more than half of the households (n=146; 56.60%). Other water treatment methods in BASECO, Manila included basic filtering with a cloth (n=63; 17.36%), sedimentation (n=6; 1.70%), and solar disinfection (n=3; 0.80%). Most households (n=312; 85.59%) owned their water source. Among the households that needed to collect water from a distance, 53 out of 142 households (37.32%) spent 1-15 minutes fetching water, 25 (17.61%) households spent 16-30 minutes, 21 (14.79%) households spent 31-60 minutes, and 16 (11.27%) households spent ≥60 minutes.

The HHI assessed toilet facilities and found that 244 (67.20%) households in BASECO, Manila used flash toilets with septic tanks. Six (1.70%) households had no toilet facilities, and six (1.70%) households used public toilets. 44 (12.12%) households shared a toilet with their neighbors. The PHI, the last of the WASH indices used in this study, revealed that most households had water sources for handwashing within their homes (n=307; 84.57%). Most households used soap when washing their hands (n=356; 98.10%) and typically washed them 6-10 times daily (n=159; 43.80%). The frequency and distribution of WASH practices in BASECO, Manila, are presented in Table 2.

**Table 2:** Frequency and distribution of WASH practices in BASECO, Manila

WASH Indices	Variables	Observations	f	%
Drinking Water Index (DWI)	Source of drinking water	Canister purified/distilled water	237	65.30%
		Tap or piped water	120	33.10%
		Underground well	3	0.80%
		Others (specify)	3	0.80%
	Drinking water treatment	Boiling	146	40.20%
		Filtering using a cloth	63	17.36%
		Solar disinfection	3	0.80%
		Sedimentation	6	1.70%
		Others	40	10.00%
		Did not treat water	105	28.90%
	Shared sources of water	Yes	51	14.05%
		No	312	85.95%
	Time to obtain water (roundtrip)	Does not collect	221	60.88%
		1 - 15 minutes	53	14.60%
		16 - 30 minutes	25	6.89%
		31 - 60 minutes	21	5.79%
		60 minutes and above	16	4.41%
		Do not know	27	7.44%
Household Hygiene Index (HHI)	Type of toilet facility	Piped sewer system	48	13.20%
		Flush toilet with septic tank	244	67.20%
		Pit latrine	37	10.20%
		I do not know	15	4.20%
		No facility	6	1.70%
		Public toilet	6	1.70%
		Others	7	1.90%
	Shared toilet facility	Yes	44	12.12%
		No	319	87.88%
	Location of the toilet facility	In own dwelling	330	90.91%
		Outside of house	33	9.09%
Personal Hygiene Index (PHI)	Availability of handwashing station	Within the household	307	84.57%
		Outside the household	29	7.99%
		Stocked bucket water	24	6.61%
		No area for handwashing within the vicinity of home/land property	2	0.83%
	Using soap for handwashing	Yes	356	98.10%
		No	7	1.90%
	Frequency of handwashing	Always (6-10 times a day)	202	55.65%
		Sometimes (1-5 times a day)	159	43.80%
		Never	2	0.55%

We further analyzed the WASH indices to understand the overall WASH condition in BASECO, Manila. The DWI covered four variables: source of drinking water, drinking water treatment, shared sources of water, and time to obtain water. From the household responses to the questionnaire, we identified the positive observations from the variables from each household. We assigned a score of 1 for every positive observation and the expected maximum score for DWI is 4. 19.01% of the households got a perfect score of 4, while 4.68% got a 0.

**Figure 2:**
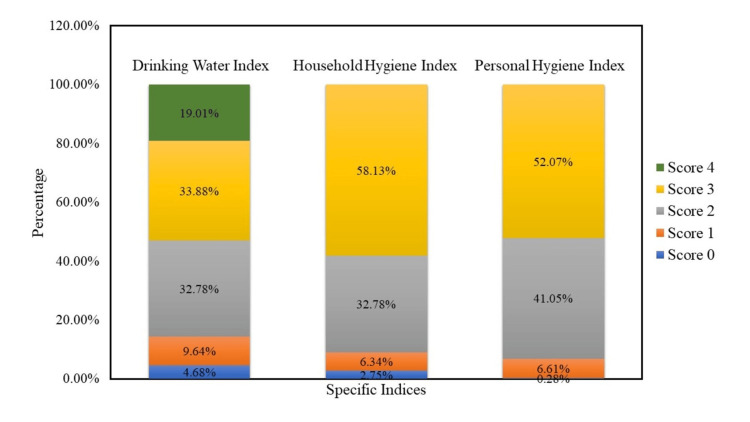
Distribution of scores in Drinking Water Index (Maximum Score =4), Household Hygiene Index (Maximum Score=3), and Personal Hygiene Index (Maximum Score=3). The x-axis reflects the percentage of the participants' score per WASH index in the y-axis. The data labels reflect the percentage of respondents who got the same number of correct scores per WASH Index.

The HHI included three variables: the type of toilet facility, shared toilet facility (yes/no), and location of the toilet facility. The HHI has a maximum total score of 3, obtained by 58.13% of the respondents. On the other hand, the PHI covered three variables measured: availability of handwashing facility, use of soap in handwashing, and frequency of handwashing. Most households (52.07%) got a perfect score of 3. Figure 2 shows the distribution of the scores across three indices of WASH.

Knowledge and risk perceptions on parasitic disease transmission

A poor WASH condition increases the risk of parasitic infections. Conversely, a high awareness of parasitic infections can help prevent individuals from contracting the disease [13]. This study also assessed the knowledge and risk perceptions concerning parasitic infections among residents of BASECO, Manila. Table 3 shows the respondents' understanding of the risk factors and symptoms associated with parasitic infections. Most respondents knew drinking unfiltered water could result in parasitic infections (n=318; 87.6%). They also recognized that improper food washing (n=309; 85.1%), using contaminated water sources (n=301; 82.9%), and consuming raw or undercooked meat (n=298; 82.1%) could lead to infections. Regarding symptoms of parasitic infections, 316 (87.1%) respondents identified diarrhea as the most common symptom, while constipation was the least recognized (n=287; 79.1%). Table 3 presents the detailed outcomes of assessing respondents' knowledge regarding parasitic infections.

**Table 3:** Assessment of the respondent’s knowledge regarding parasitic disease

Respondent's knowledge of parasitic disease		Correct Response		Incorrect Response	
		f	%	f	%
Do you know you can be infected with parasites when…?	Drinking unfiltered water	318	87.6	45	12.4
	Utilizing a contaminated water source	301	82.9	62	17.1
	Not washing food properly	309	85.1	54	14.9
	Consuming raw or undercooked meat/seafood	298	82.1	65	17.9
	Handling food with dirty hands	296	81.5	67	18.5
Do you know this is a symptom of parasitic infection?	Abdominal or stomach pain	311	85.7	52	14.3
	Diarrhea	316	87.1	47	12.9
	Nausea or vomiting	291	80.2	72	19.8
	Weight loss	293	80.7	70	19.3
	Bloating	294	81.0	69	19.0
	Constipation	287	79.1	76	20.9

Regarding risk perception, most respondents considered parasitic infections to be a severe health issue in general, with a likelihood of occurrence (Rating=4). A total of 122 respondents (33.61%) shared this view. However, they considered it a moderate concern in BASECO (Rating=3). They believed that the risk of contracting parasitic infections without preventive measures is high (Rating=4) but they viewed their current practices as providing moderate protection against parasitic infections (Rating=3). The overall perception of the seriousness of parasitic diseases among residents in BASECO, Manila was moderate, with a weighted mean score of 3.35.

This study also examined the perceived susceptibility of household members to parasitic infections. Most respondents rated it a severe concern when asked about the likelihood of contracting parasitic infections (Rating=4). However, the overall perception score was neutral (Rating=3). On another note, respondents expressed a high level of agreement (Rating=4) when asked about the potential for parasitic infections based on the quality of their drinking water. Table 4 presents the complete set of perception measures and corresponding scores provided by respondents.

**Table 4:** Risk perceptions of the respondents on parasitic diseases * With a Likert scale of 1. Very unlikely, 2. Unlikely, 3. Neutral, 4. Likely, 5. Very likely ** With a Likert scale of 1. Strongly disagree, 2. Disagree, 3. Neutral, 4. Agree, 5. Strongly agree.

Perceptions	Perception Measures	Likert Scale (*f*)					Weighted Mean	Verbal Interpretation
		1	2	3	4	5		
Perception of the Seriousness of Parasitic Diseases*	How serious do you think parasitic infections are?	71	25	75	122	70	3.26	Neutral
	Do you think you or your household members may contract parasitic infections if you do not take any preventive measures?	46	31	55	120	111	3.6	Likely
	What do you think is your or your household members' chance of contracting parasitic infections?	78	56	57	108	64	3.07	Neutral
Perception of susceptibility to parasitic infection and extent of anxiety**	Do you think you will have a parasitic infection based on the quality of your drinking water?	25	11	65	61	201	4.11	Agree
	Could you have parasitic infections in the next year?	65	72	61	107	58	3.05	Neutral

Relationships between the WASH indices, sociodemographic characters, and the knowledge and risk perceptions on parasitic diseases

The relationship between the WASH indices and their contributing factors was analyzed. The overall WASH score was first determined by aggregating the three indices, DWI, HHI, and PHI, for inclusion in the regression model. The results show that DWI has a positive relationship with the number of household members (B=0.191; p-value=0.001), PHI has a positive relationship with the respondents' knowledge of parasitic infections (B=0.112;p-value=0.047), and their age (B=0.123; p-value=0.027). The overall WASH score positively correlates with household income (B=0.105; p-value=0.045).

**Table 5:** Linear regression analysis of the relationship between the WASH indices, sociodemographic characteristics, knowledge, and risk perception on parasitic diseases DWI= Drinking Water Index; HHI= Household Hygiene Index; PHI= Personal Hygiene Index; WASH= overall WASH index *P-value ≤ 0.05; **P-value ≤0.01

Variables		DWI	HHI	PHI	WASH
Knowledge	Standard coefficient Beta	-0.34	0.054	0.112	0.082
	P-value	0.569	0.374	0.047*	0.123
Risk perception	Standard coefficient Beta	0.047	-0.016	-0.034	0.006
	P-value	0.433	0.786	0.549	0.906
Age	Standard coefficient Beta	-0.082	0.048	0.123	0.044
	P-value	0.168	0.422	0.027*	0.408
Number of household members	Standard coefficient Beta	0.191	-0.075	0.242	0.064
	P-value	0.001**	0.210	0.231	0.224
Household income	Standard coefficient Beta	0.028	0.088	0.017	0.105
	P-value	0.642	0.143	0.765	0.045*

## Discussion

Socioeconomic disparity in BASECO, Manila, was associated with overall household WASH practices

Around 70% of households in BASECO, Manila had a monthly income of PHP 10,000 or lower (≤178.18 US dollars). In 2018, there was a debate in the Philippines about the minimum monthly income needed for a family of five, following a statement by government economists that PHP 10,000.00 was enough. This statement was later corrected to PHP 42,000.00, which has a significant difference of 320% [24]. According to the PSA report in 2021, the average monthly income for Filipino families was approximately PHP 25,000.00 [25], which is significantly higher than the income levels in BASECO in 2023. Although there is no recent data from the PSA, it suggests a notable socioeconomic disparity in BASECO regarding household incomes.

Socioeconomic status plays a significant role in influencing health outcomes through various factors such as environmental exposure, health behavior, and access to healthcare. Previous research has shown that there is a strong correlation between low socioeconomic status and an increased risk of parasitic infections [26]. This is mainly attributed to the limited availability of WASH facilities, healthcare services, and vector control measures for individuals with lower socioeconomic status [5].

In this particular study, the relationship between household income and overall WASH practices is explored. The findings indicate that there is a 10.50% improvement in WASH practices for every increase in income level. However, it is important to note that this study is cross-sectional in nature, and therefore, further research is needed to strengthen the association by considering the temporal sequences of events and capturing dynamic processes over time through longitudinal studies. The study also reveals that nearly half of the respondents require improvements in household and personal hygiene practices, while over 80% of them need better access to safe drinking water.

Interventions aimed at addressing socioeconomic disparities are crucial to improving WASH practices in BASECO. This, in turn, will help prevent the transmission of parasitic infections in the community. While poverty alleviation is a complex approach, community leaders in BASECO can implement simpler interventions. One potential intervention is promoting sanitation and hygiene through communication, education, and public awareness. This study demonstrates that knowledge about parasitic diseases is positively correlated with personal hygiene, which can be particularly beneficial for community members with low educational attainment. Engaging communities in the planning, implementation, and monitoring of WASH interventions, to ensure cultural appropriateness and sustainability, can empower them to protect themselves from parasitic diseases. By taking ownership of their health, communities can achieve more effective outcomes. It is important to note that these interventions can be implemented even in the face of persistent challenges in providing subsidized or free healthcare services, as well as improving WASH facilities and infrastructure.

Aside from household income, employment status, and educational attainment disparities were evident in BASECO, both recognized as significant predictors of health outcomes in various studies [27].

Overcrowding of household members has a positive relationship with the DWI score

Household overcrowding was prevalent in BASECO, with 54.27% of the respondents reporting five to eight household members. The average size of houses in BASECO was not measured in this study, which could be important data to establish the extent of overcrowding. However, qualitative observations made by the researchers during the survey, along with the quantitative data showing five to eight members per household, served as the basis for this claim. This is a weakness and limitation of the study, but it is an interesting point for discussion.

Some studies provide statistical evidence regarding the correlation between household overcrowding and an increased risk of close contact infectious diseases [28]. Although infectious diseases are commonly associated with illnesses such as chickenpox, the common cold, and diphtheria, parasitic infections can also spread through close contact. One common mode of parasite transmission is fecal-oral transmission, which occurs from person to person. Inadequate WASH conditions facilitate this type of transmission. Parasites like *A. lumbricoides*, *T. trichiura*, and *Giardia lamblia* can infect humans through this route [10].

This study found that as the number of household members in BASECO increased, there was a notable change in the DWI score. The DWI evaluates various factors, including the source of drinking water, water treatment, shared water sources (yes/no), and the time taken to access water. The association between these variables can be attributed to the perceived susceptibility to parasitic infections. When asked about their perceived risk of parasitic infections based on the quality of their drinking water, most respondents expressed concern. The subjective belief of being at risk of a specific disease can influence individuals to adopt personal preventive measures [29]. Given the positive relationship between household overcrowding and DWI, respondents may view household overcrowding as a risk factor and take steps to enhance water quality through filtration and other treatments for preventive purposes. Although this study does not definitively prove this relationship, some studies support the idea of health behavior modifications [29]. The concept of health behavior modifications can be applied to develop intervention programs that focus on promoting sanitation and hygiene, improving health education and awareness, as well as enhancing community empowerment and participation.

Personal hygiene practices are expected to improve in people with increased knowledge of parasitic infection

This study did not assess the health information campaigns available in BASECO, Manila. This information can be useful in gaining a deeper understanding of the determinants of poor WASH practices in the community. It can also help measure the extent of willingness to improve the WASH practices of households in the community. However, it is well-known that the World Health Organization and the Department of Health in the Philippines have various global and national programs that educate the public about WASH, parasitic infections, and disease prevention methods. In addition, the Philippine Red Cross implemented a program aimed at improving access to safe water and basic sanitation in Tondo, Manila, which is a densely populated urban poor community adjacent to BASECO [15].

This study supports the importance of various educational interventions, especially those that introduce people to various agents of infectious diseases, in promoting proper WASH practices. Based on the evidence, an increased level of knowledge on parasitic infections improves personal hygiene by 11.2%. This is the same reason why WASH practices are introduced as early as childhood in schools and within the community [30]. Numerous studies have demonstrated the beneficial impact of health education on community health outcomes [26]. The positive relationship between personal hygiene practices in BASECO, Manila, and the increase in PHI scores suggests the relevance and potential effectiveness of health education campaigns in urban poor communities in Metro Manila, such as in BASECO Compound. To note, the survey scores assessed in this study were based on self-reported data, which may be influenced by factors such as social desirability, memory recall, and misinterpretation of questions. Further studies are needed to bolster the research findings and inform policy-making efforts aimed at improving the welfare of individuals in urban settings.

## Conclusions

The WASH practices in BASECO, Manila require improvement, particularly in terms of access to safe drinking water. Ensuring proper WASH practices in the area can be challenging due to limited household income, disparities in educational attainment, household overcrowding, and a high unemployment rate. The residents' personal hygiene in BASECO is noticeably good and is associated with their knowledge of parasitic infections.

In general, the people in BASECO have a good understanding of how parasites are transmitted and the symptoms of infection. However, there is a need for education on this subject for several individuals in the area. In terms of their perception of risk, they are neutral about the possibility of contracting parasitic infections in their community. However, they are concerned about their immunity based on the quality of water available to them. Several factors have emerged as instrumental in their WASH practices, including risk perception regarding parasitic diseases, socioeconomic disparities, and household overcrowding. These factors play a crucial role in identifying areas for improvement and promoting health policies for urban poor communities in the Philippines.
